# Tsallis Entropy for Cross-Shareholding Network Configurations

**DOI:** 10.3390/e22060676

**Published:** 2020-06-17

**Authors:** Roy Cerqueti, Giulia Rotundo, Marcel Ausloos

**Affiliations:** 1 Department of Social and Economic Sciences, Sapienza University of Rome, p.le A. Moro 5, 00185 Roma, Italy; roy.cerqueti@uniroma1.it; 2 School of Business, London South Bank University, London SE1 0AA, UK; 3 Department of Statistical Sciences, Sapienza University of Rome, p.le A. Moro 5, 00185 Roma, Italy; 4 School of Business, College of Social Sciences, Arts, and Humanities, Brookfield, University of Leicester, Leicester LE2 1RQ, UK; marcel.ausloo@ulg.ac.be; 5 Group of Researchers for Applications of Physics in Economy and Sociology (GRAPES), Rue de la belle jardinière, 483, Sart Tilman, B-4031 Angleur, Liege, Belgium; 6 Department of Statistics and Econometrics, Bucharest University of Economic Studies, Calea Dorobantilor 15-17, 010552 Sector 1 Bucharest, Romania

**Keywords:** Tsallis entropy, copula functions, cross-shareholding network, finance

## Abstract

In this work, we develop the Tsallis entropy approach for examining the cross-shareholding network of companies traded on the Italian stock market. In such a network, the nodes represent the companies, and the links represent the ownership. Within this context, we introduce the out-degree of the nodes—which represents the diversification—and the in-degree of them—capturing the integration. Diversification and integration allow a clear description of the industrial structure that were formed by the considered companies. The stochastic dependence of diversification and integration is modeled through copulas. We argue that copulas are well suited for modelling the joint distribution. The analysis of the stochastic dependence between integration and diversification by means of the Tsallis entropy gives a crucial information on the reaction of the market structure to the external shocks—on the basis of some relevant cases of dependence between the considered variables. In this respect, the considered entropy framework provides insights on the relationship between in-degree and out-degree dependence structure and market polarisation or fairness. Moreover, the interpretation of the results in the light of the Tsallis entropy parameter gives relevant suggestions for policymakers who aim at shaping the industrial context for having high polarisation or fair joint distribution of diversification and integration. Furthermore, a discussion of possible parametrisations of the in-degree and out-degree marginal distribution—by means of power laws or exponential functions— is also carried out. An empirical experiment on a large dataset of Italian companies validates the theoretical framework.

## 1. Introduction

The presence of interconnections among companies is the ground for the propagation of shocks over the entire industrial structure of a country; see e.g., [1,2]. This evidence has led to a growing number of studies exploring such structure through networks theories; see e.g., [3,4].

In this respect, a single company can be intuitively seen as a network node. The ownership relationship can be represented through a network: there is a (directed) link from a company *i* to a company *j* if *i* holds shares of *j*. For what concerns the mutual connections among companies, several contexts can be explored on the basis of the topic under investigation. Here, we mention connections that are driven by technological transfer [5], the presence of personal relationships [6,7,8], the interlock of directorates [9,10,11,12], and capabilities at the organisational level [7,13]. For a survey on this field, see e.g., [14].

We propose a specific focus on the cross-shareholding matrix, which is associated to the directed links, thereby capturing the so-called in-degree and out-degree of each node.

Specifically, the in-degree of a company—say, $k_{in}$—is the number of companies holding some ownership of the considered node. Such a concept has a clear interpretation on the integration of any given company in its reference industrial and productive environment. Similarly, the out-degree of a company—namely, $k_{out}$—counts the companies included in the portfolio of the considered node. Thus, $k_{out}$ is associated to diversification, which, in turn, might point to information on the possible reaction of a considered company to markets fluctuations. For the concepts of integration and diversification, we refer the interested reader to [15].

Notice that the so-followed approach is grounded on the existence of a connection—in terms of ownership relations—between two companies. In so doing, we explore diversification and integration— along with market concentration, which is a synthesis of them—as a matter of pure shareholding strategies and through the singular attitude of companies to collect shares of other companies, and at the same time to have shares own by other companies—“other companies”, which can be the same being owner and owned (Renault SA, which is part-owned by the French state, owns 43% of Nissan Motor Co, while the Japanese firm has 15% of the French carmaker—but with no voting rights in this case). Within such thinking, the amount of inter company flows leads to a discussion on the size of the connections between companies. In this setting, in-degrees and out-degrees should be reasonably written as sums of percentages of in-flows and out-flows. Thus, the in-degree can be high in both cases, i.e., when there is a large number of existing in-connections with small flows or small values of in-connections with large entities of flows; the same consideration applies also for the out-degree, of course. The numerical dimension of the connections is then lost—even if a new information on the size of the flows is available. Yet, the analysis of such flows is clearly beyond the scopes of the present paper.

While out-degrees are widely explored, for their natural connections with the resilience of an industrial system, see e.g., [16,17,18,19,20,21], scarce attention has been paid to in-degrees. Let us point to a noticeable contribution on the trade-off between diversification and integration in the analysis of economic crises in [22].

Here, we are concerned by the market concentration—which represents a synthesis of diversification and integration, by means of the entropy of the in-degree and out-degree distributions. The entropy concept allows for understanding the position of the considered industrial structure between the extreme cases of uniform diversification and integration and *a contrario* strong polarisation, with only one company playing the role of the leader.

Furthermore, we also include a deep analysis of the particular features of the distributions through a generalised concept [23,24] of Boltzmann–Gibbs (or equivalently Shannon information [24,25,26]) entropy. To this end, we move from [27] and deal with the Tsallis entropy for discussing the in- and out-degrees distributions of the companies.

Tsallis entropy—introduced in [23]—has been applied in a number of contexts related to economics and finance; see e.g., the excellent review in [28] and references therein. Most of the time, the studies concern risk or portfolio management [29,30,31,32,33,34]. Our present report seems to be the first contribution dealing with this powerful instrument in the context of the cross-shareholding matrix for its related network of companies.

Tsallis entropy depends on a (usually real, see a complex case in [35]) parameter, whose interpretation provides relevant information on the shape of the distributions. Indeed, when the parameter is negative (positive), then Tsallis entropy attains its maximum in the highest polarisation case (in the uniform distribution case). Moreover, a negative value of the parameter is associated to a strong relevance of fat tails and rare events; see e.g., [24].

To explore in depth the relationship between integration and diversification, we propose the analysis of the joint distributions between such terms in the relevant cases of independence—i.e., when the stochastic dependence is described by a product copula—and in the maximum level case of positive (negative) dependence—i.e., when the dependence is given by the upper (lower) Frechet bounds copulas. These represent the mathematical bounds of the set of the copulas corresponding to the cases of perfect positive (negative) correlation; see [36]. For a complete description of the concept of copulas and on how it serves as modelling stochastic dependence, see e.g., [37,38] and refer to the Sklar’s Theorem [39]. Indeed, Sklar’s Theorem provides a reading of the copulas as mathematical functions transforming the marginal distributions of a set of random variables into their joint distribution (see also below).

We consider a high-quality dataset of holdings listed in the Italian Stock Market to validate our theoretical proposal. Such a selection, the Italian Stock Market as reference context, has been driven by data availability. Indeed, the phase of data collection has been particularly challenging, with manual collection procedures and matching among different datasets—see the details in Section 4.1. Of course, data availability is the premise of the data collection procedure. This said, even if it is theoretically easy to reproduce the analysis for all the major markets—like the US and the UK ones, the practical implementation in different contexts requires a non-trivial effort and data availability.

We also propose an extension of the analysis to a wide and universal economic system, where in-degrees are assumed to be synthesised by two parametric functions of either power law or exponential types, while the out-degree distribution obeys a power law; see e.g., [40]. In particular, we have included the parameters of such functions in the calibrating quantities set. Such a proposed extension leads to useful discussions about the assessment of missing links in the cross-shareholding matrix, in line with some literature contributions, like e.g., [41,42,43,44,45,46].

Some results emerge from our study. The obtained outcomes suggest strategies that should be implemented by policymakers if pursuing a highly polarised industrial structure goal—with a company holding the shares of all the other ones and, at the same time, whose shares are included in the portfolios of the others—or a fair joint distribution of diversification and integration. Such policies are built on the basis of the dependence structure between in-degrees and out-degrees and on enforcing the shapes of their distributions in a proper way.

The remaining part of the paper is organised as follows. Section 2 provides some information on the reference literature on cross-shareholding. Section 3 gives the details of the methodological devices used in the analysis. Section 4.1 provides a description of the dataset employed for the methodological validation and, in particular, the network construction in Section 4.2. Section 5 describes and discusses the obtained findings. Conclusions and comments on policy implications are found in Section 6.

## 2. Brief Review of the Reference Literature on Cross-Shareholdings

This section provides a list of key papers dealing with cross-shareholdings. Such a list is not exhaustive, but the referred contributions are particularly close to the present study—even if they present remarkable differences. As a premise, we have to state that the framework adopted in this paper is quite new when compared to other papers on the cross-shareholdings.

In [47], a complex networks approach is used for identifying the companies that are central in the information flow and for the control. The coupling among in-degree and out-degree is not examined explicitly, although it intervenes in the empirical estimates of the flow-betweenness and of other centrality measures.

The perspective in Abreu et al. [48] is of an empirical nature, without a precise focus on the relationship between in-degrees and out-degrees, i.e., as integration and diversification measures, respectively.

In [49], the possibility to use cross-shareholdings for achieving the control of companies through intermediaries is examined, but there is again no deeper insight on the relationship between integration and diversification as optimal means toward the considered specific targets.

Vitali et al. [50] offer the analysis of the structure and topology of the transnational ownership network of cross-shareholdings. This is a pretty empirical paper, without further steps in the analysis of the stochastic dependence on integration and diversification.

An analysis of the relevance of the cross-shareholdings in the Japanese markets can be found in [51]. The target of the quoted paper is to understand the role of shareholdings in order to reduce the risk/performance ratio. However, the aim is quite different from the one tackled in this present paper.

In [52], Okabe performs an economic analysis on cross-shareholdings in Japan, where this theme is quite relevant. Trends and implications for the Japanese economic system and related public policies are discussed. In [53,54] the focus is on the presence of the power law, and [55] adds more insights typical of complex networks studies. However, such analyses are mostly performed from the perspective of economics and empirical investigation rather than by proposing novel methods.

The framework of the stochastic dependence among integration and diversification considered in the present paper is close to that in [56], but presently under a wider viewpoint; in [56], one uses a rewiring procedure as methodological instrument.

## 3. Methodology

This section describes the techniques and the tools used for achieving the targets of the analysis.

### 3.1. Preliminaries and Notations

First, we introduce the main concepts that are used in the paper.

Given a node j∈V, the in-degree $k_{in}\left(j\right)$ represents the integration, i.e., the number of companies owning shares of company *j*. It is defined as follows:

$$k_{in}\left(j\right)=\sum_{i=1}^{N}a_{ij}$$

In the same line, given i∈V, the out-degree $k_{out}\left(i\right)$ represents the diversification, i.e., the number of companies in the portfolio of company *i*. It is defined as follows:

$$k_{out}\left(i\right)=\sum_{j=1}^{N}a_{ij}.$$

$k_{in}$ and $k_{out}$ both have to be considered here as random variables, whose empirical distributions are obtained by considering the real data described in Section 4.1.

The cumulative distribution functions of $k_{in}$ and $k_{out}$ is denoted by $F_{k_{in}}:R\to \left[0,1\right]$ and $F_{k_{out}}:R\to \left[0,1\right]$, respectively. Their joint distribution is denoted by $F_{k_{in},k_{out}}:R^{2}\to \left[0,1\right]$.

The generic joint distribution function $F_{k_{in},k_{out}}$ is associated to a bivariate density function. It is discrete, in the empirical case we are treating; the distribution is denoted by $p=(p_{ij}:i=1,\ldots ,n;j=1,\ldots ,m)$ such that

(1) $$p_{ij}=Prob\left(k_{in}=i,k_{out}=j\right),\;\forall \;i,\;j,$$

with

$$\sum_{i,j}p_{ij}=1.$$

The values of the integers *n* and *m* will be properly fixed in the subsequent empirical analysis.

In the sequel, for such a bivariate probability distribution, we compute the Tsallis entropy, usually defined as follows:

(2) $$S_{q}=\frac{1}{q-1}\left(1-\sum_{i,j}p_{ij}^{q}\right),$$

where q∈R is the Tsallis parameter.

A bivariate copula $C:{\left[0,1\right]}^{2}\to \left[0,1\right]$ (see e.g., [38]) is a special function that is able to describe the dependence structure between two random variables through the classical Sklar’s Theorem (see [39]). We enunciate such a crucial result by employing the notation that is used in the present paper.

**Theorem** **1.** *Sklar’s Theorem: there exists a copula $C:{\left[0,1\right]}^{2}\to \left[0,1\right]$ such that, for each $\left(s,h\right)\in R^{2}$, one has*

(3) $$F_{k_{in},k_{out}}\left(s,h\right)=C\left(F_{k_{in}}\left(s\right),F_{k_{out}}\left(h\right)\right).$$

*If $F_{k_{in}},F_{k_{out}}$ are continuous, then C satisfying (3) is unique. Conversely, if C is a copula and $F_{k_{in}},F_{k_{out}}$ are distribution functions, then $F_{k_{in},k_{out}}$ in (3) is a bidimensional joint distribution function with marginal distribution functions $F_{k_{in},k_{out}}$.*

According to Theorem 1, copulas describe different types of stochastic dependence that could be found between two random variables. In so doing, one is also capable of providing insights on the nature of the stochastic dependence of tis empirical joint distribution.

We denote by $F_{k_{in},k_{out}}^{C}:{\left[0,1\right]}^{2}\to \left[0,1\right]$ the joint distribution function resulting from the application of Sklar’s Theorem with a generic copula *C*, according to the previous Formula (3).

#### Reasoning behind the Tsallis Entropy

This section is devoted to the justification of the selection of Tsallis entropy as a key methodological measurement device. We provide a comparison between Tsallis entropy and the well-known and largely used Gibbs entropy. In fact, Tsallis entropy is known to exhibit substantial strengths when compared to the Gibbs one. To support this statement, we proceed under both technical and applied perspectives.

From a purely mathematical point of view, Tsallis entropy represents a generalisation of the Gibbs entropy. Indeed, Tsallis entropy, formally a fractional exponential approach, depends on an often real (but see [35]) parameter *q*, introduced in Equation (2); when q→1, the Tsallis entropy collapses to the Gibbs entropy. Hence, the Tsallis entropy is able to capture several aspects that are not covered by the Gibbs entropy—all of those aspects related to a not unitary parameter *q*. In our context, the main results will be seen to be related to negative *q* values. Thus, it is clear that the Gibbs entropy would not allow us to provide a deep understanding of the nature of the stochastic dependence between in-degree and out-degree distributions.

In the context of applied science, we may recall that classical statistical mechanics of macroscopic systems in equilibrium is based on Boltzmann’s principle and Gibbs entropy. However, Boltzmann–Gibbs statistical mechanics and standard thermodynamics present serious difficulties or anomalies for non-equilibrium, open, non-ergodic, non-mixing, systems, and for those that exhibit memory retention. Within a long list, we might mention systems that involve long-range interactions (see e.g., [57,58]), non-Markovian stochastic processes, like financial markets (see e.g., [59,60,61,62,63,64]), dissipative systems in a phase space that has some underlying looking (multi)fractal-like structure (see e.g., [65]), like many open social systems, all hardly having an additive property (see e.g., [66]).

In brief, Tsallis theory provides a better thermo-statistical description than the standard Boltzmann– Gibbs formalism, because the Tsallis fractional exponential approach allows for encompassing cases of non-equilibrium and dissipative systems into hard core statistical mechanics principles.

### 3.2. Outline of the Analysis

The analysis is carried out in two main directions.

First, we compute and discuss the Tsallis entropy of the joint distribution $F_{k_{in},k_{out}}^{C}$, which is obtained by applying the Sklar’s Theorem with some specific copulas *C*. In so doing, we provide useful insights on the behaviour of the cross-shareholding system under different scenarios of interactions between in-degrees and out-degrees.

In particular, we address the corner cases of maximal positive and negative dependence, and the case of independence. Such cases correspond to the following copulas:

- Product (independence)
  (4) $$C_{P}\left(u,v\right)=uv$$
- Lower Frechet (maximal negative dependence) and Upper Frechet (maximal positive dependence)
  (5) $$C_{LF}\left(u,v\right)=\max\left\{u+v-1,0\right\},\;C_{UF}\left(u,v\right)=\min\left\{u,v\right\}.$$

Second, we discuss the sensitivity analysis of the in- and out-degrees distributions when they are properly parametrised, by means of the Tsallis entropy.

In this respect, while the literature points out the ubiquitous presence of a power law for the out-degree distribution, the in-degree is much less studied. However, the main theoretical functions that can be suitably used for approximating the in-degree empirical distribution are either the power law or the exponential law (see [27] and references therein contained). Therefore, on one side, we consider the marginal distribution of the out-degree as following a power law; on the other side, we consider two cases, power law or exponential function for modelling the in-degree empirical distribution.

The power law and the exponential law for a generic discrete random variable *X* are defined, as follows:

- Power law:
  (6) $$Prob\left(X=x\right)=ax^{-k},$$
  where x≥0, a>0 is a normalising constant and k>0.
- Exponential law:
  (7) $$Prob\left(X=x\right)=ae^{-kx},$$
  where x≥0, a>0 is a normalising constant and k>0.

Thereafter, we implement the sensitivity analysis in three cases:

(A) under the hypothesis of $k_{out}$ described by a power law as in (6) and $k_{in}$ has its empirical distribution, the power law exponent *k* is allowed to change and is treated as a parameter;
(B) under the hypothesis of $k_{in}$ power law as in (6) and $k_{out}$ empirical: the power law exponent *k* is allowed to change and is treated as a parameter; and,
(C) under the hypothesis of $k_{in}$ exponential as in (7) and $k_{out}$ empirical: the parameter *k* in the exponential is allowed to change, as any parameter does.

Thus, in each case, there are two parameters: *q* for the Tsallis entropy and *k* for the power law or exponential. In all cases, we have employed the three copulas $C_{I}$, $C_{LF}$, and $C_{UF}$ introduced in (4) and (5) for deriving the joint probability distribution, according to Theorem 1.

## 4. The Network

Here, we present the cross-shareholding network that are used in the analysis.

### 4.1. The Data

We consider the data already used in [27,67]. The dataset gathers data of the Milan Stock Exchange (MIB30) on 10 May 2008. First, data were obtained through the CONSOB database. For each company *j*, an informative page is shown, which contains the information on the holdings, which is the list of companies *i*, traded in the same market, which the shares of *j* are sold to. The set of all of the couples (i,j) constitutes the matrix of cross-holdings. CONSOB is the major surveillance body for the Italian Stock Market. CONSOB verifies the transparency of market operations; it has the power to stop the market in case of excess of losses/returns; CONSOB controls the proper disclosure of information. Unfortunately, only CONSOB records the holdings above 2%. Therefore, the data were cross-checked through the Bureau Van Dijk platform.

Differently from the database of prices of the shares, there is no command that allows for downloading all of the data at once. The data gathering requires manual opening of each file, and manual storing of the relevant information. Moreover, the way in which the companies are named is not uniform: sometimes, shortcuts are used instead of the original extended names. Therefore, the data collection cannot be done automatically “blind folded”. The data also have to be gathered at a selected date: it is like taking a picture of the actual situation of the market on a specific day. The time needed for gathering the data and finalising the sample is quite long, since the data were manually cross-checked with other databases. Notice that the data on banks were cross-checked with the BANKSCOPE database, which, as the name suggests, is specifically focusing on banks, hence not reporting data on other companies.

On the other side, AIDA provides some complementary information, since AIDA contains information on all companies—apart from banks. The cross-checking was necessary to be sure that we include in the database all ownerships due to investments and all cross-relationships among companies—yet excluding some very minor ones due to the management of portfolios by mutual funds. Alas, some companies had very incomplete data. Finally, the resulting sample contains the cross-holdings of 247 stocks of companies. They represent 94% of the total amount of MTA segment (MTA stands for Borsa Italiana’s Main Market, that is Italian Main Stock Market. MTA is a regulated market subject to stringent requirements in line with the expectations of professional and private investors.). The sample corresponds to 95.22% of the total capitalisation on that date, May 10th, 2008, which nevertheless makes the analysis quite suitable for a whole outlook about the links among the most relevant traded companies. Notice that the total number of cross-ownership is 243, thus less than the number of companies. In fact, there are companies traded in the Italian Stock Market, which do not buy or sell shares of other companies traded in the Italian market.

The vast majority of holdings is due either to industrial purposes or to an internal organisation of companies: for instance, the energy company ENI owns shares of two other companies, SAIPEM and SNAM RETE GAS, with a specific focus on gas delivery management. Another example is given by the financial company IFIL, which is managing the financial parts of FIAT (now merged in FCA) and JUVENTUS (football club). In turn, IFI PRIV owns the “privileged” part of IFI, belonging to the Agnelli family.

The number of companies holding shares of *k* other companies decreases sharply as *k* increases. In fact, there are 72 companies owning shares of only one other company; 16 companies owning shares of two other companies; only seven and six companies are owning shares of three and four other companies, respectively. There are only zero or one companies holding shares of six or more other companies; the maximum ownership in 19 companies is due to the insurance company “Assicurazioni Generali”, which uses ownership as part of its institutional mission. The clear prevalence of shareholders who hold shares of only one or a few other companies has been detected in other datasets [68,69].

A symmetric question holds: which is the number *h* of companies to which a specific company has sold shares? According to the literature on this topic, the question is less popular than the previous one. In our specific dataset, the maximum value of *h* is 10; there are 84 companies that sell their shares to only one company; 29 companies sell their shares to two companies; 15 are selling to three; only five companies have sold to four other companies, and another five are selling to more than four companies. Therefore, roughly speaking, the very prevailing behaviour is the relation through a sale of shares to only one other company in the market.

### 4.2. Construction of the Network

The firms are represented by the nodes of an unweighted network. We collect them in a set V={1,⋯,N}. If a company *j* is held by company *i*, then there is a directed link from *i* to *j*. The links are collected in a set *E*. In so doing, we create a network (V,E), whose adjacency matrix $A={\left(a_{ij}\right)}_{i,j\in V}$ is a N×N matrix, such that $a_{ij}=1$ if (i,j)∈E and $a_{ij}=0$ otherwise.

The insulated nodes have been removed from the analysis; the giant component and the small connected components being kept, the network is made of 158 nodes, hence the adjacency matrix is 158 × 158.

## 5. Results and Discussion

Here, we report the results of the analysis, along with a discussion of these.

As a premise, we set n=10 and m=19, in accord to the maximum values of $k_{in}$ and $k_{out}$, which are observed in the empirical dataset.

It is immediate to observe that the Tsallis entropy $S_{q}$ in (2) is strictly decreasing with respect to the parameter q∈R, with an asymptotic behaviour being given by

$$\lim_{q\to -\infty }S_{q}=+\infty ;\;\lim_{q\to +\infty }S_{q}=0.$$

This said, we restrict our graphical representations of the behaviour of the Tsallis entropy with respect to *q* to a small interval, including zero, for a better visualisation of the outcomes.

**Figure 1:**
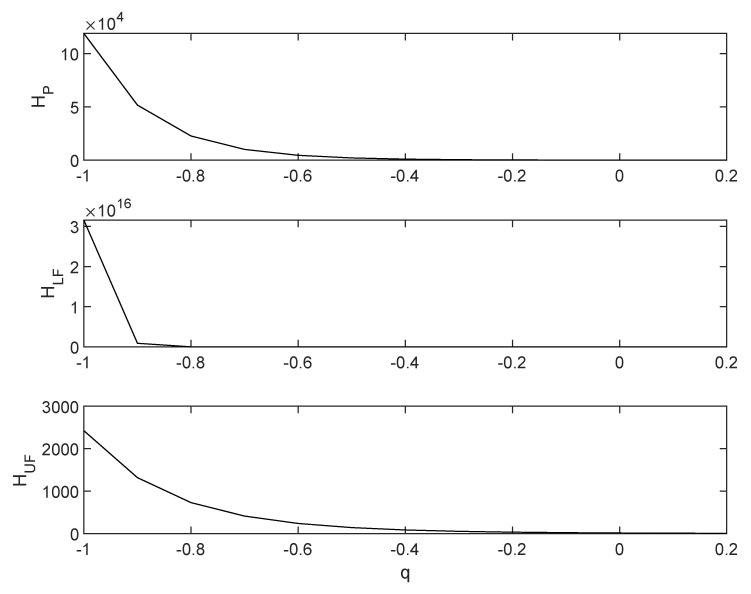
The Tsallis entropy $H=H_{P},H_{LF},H_{UF}$ as a function of *q*, in the cases of copula $C=C_{P},C_{LF},C_{UF}$ as in (4) and (5)—upper, middle and lower panel, respectively.

Figure 1 shows the behaviour of the values of the Tsallis entropy as the parameter *q* varies, in the three cases of joint distributions, $F_{k_{in},k_{out}}^{C}$ with $C=C_{P},C_{LF},C_{UF}$, as in (4) and (5)—in the upper, middle, and lower panel, respectively.

The Upper Frechet bound is the one with the slowest decrease; it is substantially flat with respect to the other cases. Moreover, the Lower Frechet bound is associated to very high values of the Tsallis entropy when *q* approaches −1; such a case is also the one presenting a very rapid collapse of $S_{q}$ as *q* increases.

An interpretation of these results is in order. The predominance—to be intended as the highest values of Tsallis entropy—of the case of copula $C_{LF}$ means that the joint probability between in-degree and out-degree is highly polarised when there is a perfectly negative correlation between such quantities. This is particularly true when *q* is negative; hence, the fat tails of the distribution do play a key role in determining such an outcome. The results change when moving to the independence and the maximum level of positive dependence. In particular, the Upper Frechet case corresponds to the highest similarity between the uniform case and the considered joint probability distribution.

The policymaker should then force the in-degrees and out-degrees of the companies to exhibit similar patterns—i.e., integration and diversification should coincide—when the target is a homogeneous industrial structure; *a contrario*, integration and diversification should be forced to exhibit a large discrepancy, if the aim of the policymaker is to foster the predominance of a company over the others.

We now deal with the cases (A), (B) and (C) described in the previous section, which are related to different parametrizations of the in- and out-degree marginal distributions.

(A) $k_{out}$ is described by a power law as in (6), while $k_{in}$ is taken with its empirical distribution.

**Figure 2:**
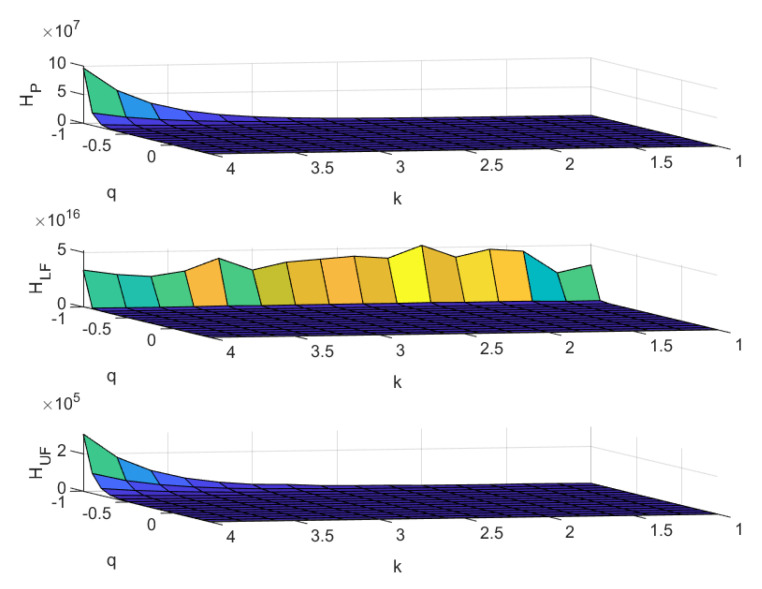
Tsallis entropy as a function of its parameter *q* and the exponent of the power law *k* for the out-degree. All the cases of copula $C=C_{P},C_{LF},C_{UF}$, as in (4) and (5)—upper, middle, and lower panel, respectively—are reported.

Figure 2 shows the Tsallis entropy as a function of its parameter *q* and the exponent of the power law *k* for the cases of copula $C=C_{P},C_{LF},C_{UF}$ as in (4) and (5)—upper, middle and lower panel, respectively.

In all cases, we observe that Tsallis entropy is decreasing as *k* decreases and *q* increases. The growth toward infinity is very rapid as *q* approaches −1. This behaviour is more evident when *k* assumes large values, i.e., when the probability that $k_{out}$ assumes a large value is particularly small—and when in-degree and out-degree are highly positively correlated or are uncorrelated. If in-degree and out-degree have the maximum level of negative correlation, then the same behaviour seems to be rather independent from the value of the power law parameter. The apparent crests on $H_{LF}$ actually correspond to very high values of $H_{LF}$; furthermore, the case with $C_{LF}$ is confirmed to have the highest level of Tsallis entropy.

We can read the results by stating that the joint probability of in- and out-degree shows a high level of polarisation in the presence of a perfectly negative correlation. Such a finding does not depend on the specific parametrisation of the out-degree through a power law. Differently, we see polarisation only for *k* large enough when the cases of stochastic independence or perfectly positive correlations are considered. This behaviour is amplified for negative *q* values, hence giving credit to the action of the fat tails of the distribution in determining it.

The policymaker has now two devices for shaping the considered industrial structure. Beyond dealing with the dependence between diversification and integration—we refer to the comments stated above for Figure 1—she/he can also force the individual companies to form specific out-degrees distributions. Indeed, in the particular cases of independence and maximum positive correlation, one can obtain some polarisation by shaping the out-degrees in order to obtain a low probability of having large values—i.e., by taking large values of the parameter *k*. Such an action is not needed when the correlation between in-degree and out-degree is of perfectly positive type.

(B) $k_{in}$ is a power law as in (6) and $k_{out}$ has its empirical distribution.

**Figure 3:**
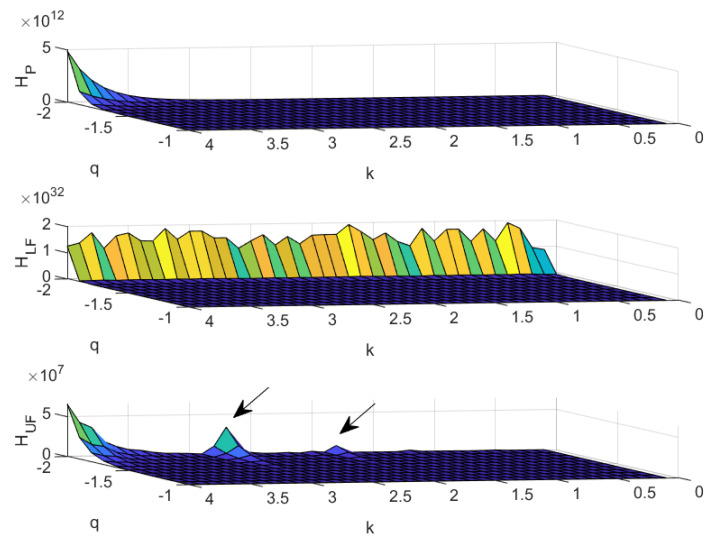
Tsallis entropy as function of its parameter *q* and the exponent of the power law *k* for the in-degree. The cases of copulas $C_{P},C_{LF},C_{UF}$ as in (4) and (5) are presented in the upper, middle, and lower panel, respectively.

Figure 3 presents the values of the Tsallis entropy as a function of *q* and *k*. Additionally, in this case, copulas $C_{P},C_{LF},C_{UF}$, as in (4) and (5), are in the upper, middle, and lower panel, respectively. For a better visualisation of the results, we only display when q<0.

The behaviour of the Tsallis entropy is quite similar to that of case (A), with four noticeable exceptions. Firstly, the scales are completely different. The values of the Tsallis entropy are much higher in this case than in case (A). Secondly, to appreciate the decreasing behaviour of the Tsallis entropy, one needs to take *q* close to −2, instead of q=−1, as in the previous case. Thirdly, we observe a deviation in the case of perfectly negative correlation, with two lines of local maxima occurring at q≃−2, for k=2.7 and k=1.8 (see the arrows in Figure 3). Fourthly, the crest appearing in the case of perfectly negative correlation is much more jagged than in case (A).

The similarities between cases (A) and (B) ensure that all of the comments raised for (A) remain valid also for this case (B). The presence of local maxima and the jagged crest do point to the questionability of the power law parameter as a device for controlling the polarisation of the joint distribution between in-degree and out-degree when the value of *q* is at its minimum. This is particularly evident for the case of perfectly negative correlation—i.e., in the case of jagged crest—while an action for properly calibrating the parameter k≃−2.7 and ≃1.8 remains possible for the case of perfectly positive correlation.

(C) $k_{in}$ has an exponential distribution as in (7) and $k_{out}$ has its empirical distribution.

**Figure 4:**
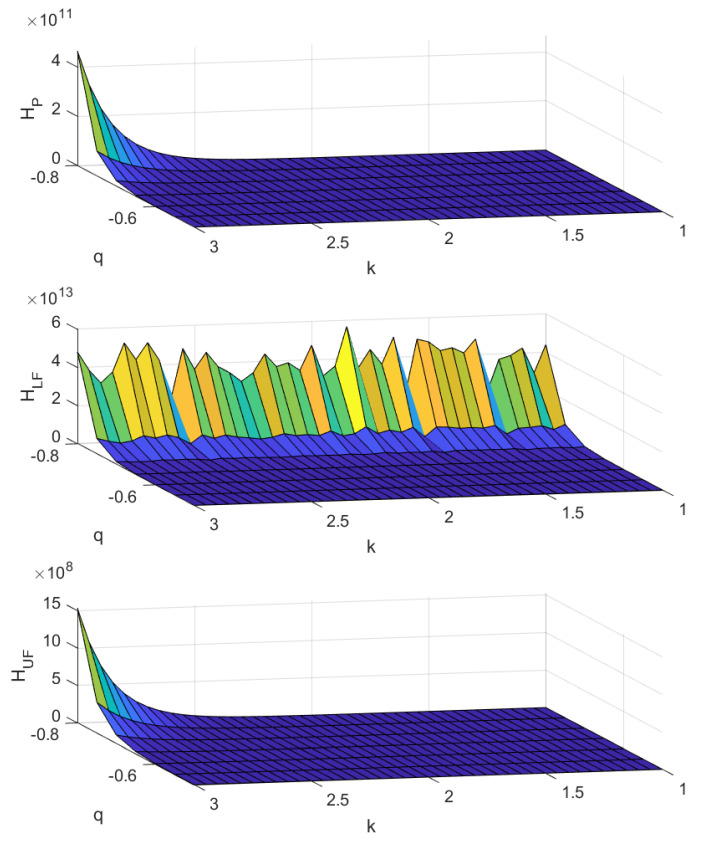
Tsallis entropy as a function of parameter *q* and *k* for describing the exponential decrease of the in-degree. The cases of copulas $C_{P},C_{LF},C_{UF}$, as in (4) and (5), are described in upper, middle and lower panel, respectively.

The upper, middle, and lower panel of Figure 4 display the Tsallis entropy as a function of *q* and *k*, for copulas $C_{P},C_{LF},C_{UF}$ as in (4) and (5), respectively; for a clear view of the behaviour of the surface, we only present q<0.

As for (B), the behaviour of Tsallis entropy is also analogous to the one observed for (A), but with three main differences. Indeed, the decreasing behaviour of the Tsallis entropy can be properly visualised for *q* close to −0.8 (it was −1 and −2 in cases (A) and (B), respectively); moreover, the crest appearing in the middle panel at low values of *q* is more jagged here than in (A); finally, the minimum value of *q* appearing in Figure 4 is −0.8 instead of −1 (case A)) and −2 (case B)).

Some relevant insights can be derived by comparing the three cases (A), (B), and (C). When the desired target is to shape the cross-shareholding network for a polarised situation—with a company holding the widest part of shares of the others and, at the same time, whose shares are in the portfolios of the other companies—then one has to impose a perfectly negative dependence between the in-degree and the out-degree. Moreover, one has also to shape the distribution of the in-degree as a power law; this means that the probability of having a high in-degree value has to be lower than that of having a low in-degree value. Lastly, the joint distribution between in-degree and out-degree should include also the presence of fat tails, so that one can employ the informative content of the Tsallis entropy in the case of large negative value of *q*. Under the conditions described above, the Tsallis entropy attains its highest value—see case (B), middle panel. Differently, by imposing the maximum level of positive dependence and a power law behaviour on the out-degree distribution, with a small value of the parameter *k*, one pursues the objective of shaping the industrial structure towards a more uniform integration and diversification; see case (A), lower panel.

## 6. Conclusions and Policy Implications

To conclude, we can offer some general remarks on policy implications.

The starting point of the analysis is to describe the industrial structure of a country—in terms of market integration and diversification and, consequently, of concentration. In this respect, the policy makers might aim at fostering the competition in the market or, conversely, at shaping the market for having a leading company.

This theme is of paramount relevance for policy makers. Indeed, the interest of regulatory authorities in the raise of concentration is witnessed by its explicit insertion in official documents. For instance, the study of the classical Herfindahl-Hirschman index (HHi)—which is a relevant measure of market concentration—plays a significant role in the assessment of possible enforcement of US antitrust laws [70]. Since 1982, the Merger Guidelines by the U.S. Department of Justice and the Federal Trade Commission [71] have provided an indication for the identification of post merger markets as “unconcentrated”, mildly concentrated, or highly concentrated based on the value of HHi. For a more scientific perspective, we refer e.g., to [56,72]. In this respect, we also mention [22], who have shown that some peculiar combinations of integration and diversification might lead industrial structures to be more vulnerable to financial fluctuations.
